# Quantitative computed tomography for assessing body composition in schizophrenia: a potential indicator of insulin resistance

**DOI:** 10.3389/fpsyt.2025.1676939

**Published:** 2025-12-08

**Authors:** Xinping Kuai, Fangsong Zhang, Mengyang Han, Jianye Zhang, Chanjun Ding, Lili Yuan, Qicheng Yuan, Lei Chen, Ziyue Xu, Xuexue Wang, Jinhong Wang

**Affiliations:** Department of Radiology, Shanghai Mental Health Center, Shanghai Jiao Tong University School of Medicine, Shanghai, China

**Keywords:** schizophrenia, quantitative computed tomography, insulin resistance, visceral adipose tissue, subcutaneous adipose tissue

## Abstract

**Objective:**

To investigate the relationship between the Homeostasis Model Assessment of Insulin Resistance (HOMA-IR) index and organ body composition, and to evaluate the potential of quantitative computed tomography (QCT) parameters as biomarkers for indicating insulin resistance (IR) in schizophrenia.

**Materials and methods:**

A total of 924 patients with schizophrenia were enrolled and categorized into non-IR (HOMA-IR ≤ 2.5) and IR (HOMA-IR > 2.5) groups. Quantitative computed tomography (QCT) systematically measured parameters such as bone mineral density (BMD), hepatic fat fraction, and areas of subcutaneous adipose tissues (SAT) and visceral adipose tissues (VAT). Univariate and multivariate logistic regression analyses were performed to identify independent predictors of IR. The indicative performance of individual parameters and combined models-including a QCT parameters-only model and a model integrating QCT parameters with clinical covariates (gender, age, BMI)-was evaluated using receiver operating characteristic (ROC) curve analysis.

**Results:**

Patients in the IR group (n=332) demonstrated significantly elevated levels of all QCT parameters compared to the non-IR group (n=592): BMD (125.27 vs. 114.01 mg/cm³), liver fat (10.14% vs. 6.61%), SAT (138.55 vs. 82.20 cm²), and VAT (222.83 vs. 131.92 cm²) (all *P* < 0.001). While all four parameters were significant in univariate analysis, multivariate analysis adjusted for age, sex, and BMI identified VAT (Adjusted OR: 1.013, 95% CI: 1.009–1.016, *P* < 0.001) and liver fat content (Adjusted OR: 1.092, 95% CI: 1.051–1.135, *P* < 0.001) as the only independent indicators. The area under the curve (AUC) for individual parameters was 0.790 for VAT, 0.769 for SAT, 0.664 for liver fat, and 0.574 for BMD. The combined QCT parameters-only model achieved an AUC of 0.834, which was significantly improved to 0.852 (*P* = 0.001 by DeLong’s test) after incorporating clinical covariates.

**Conclusion:**

Visceral adipose tissue and liver fat content, as measured by QCT, are robust and independent indicator of insulin resistance in patients with schizophrenia. A model combining QCT parameters with basic clinical covariates offers the best predictive performance for identifying IR risk in this population, highlighting the critical role of ectopic fat deposition in metabolic dysregulation.

## Introduction

Schizophrenia, a complex neuropsychiatric disorder, is increasingly emerging as a focus of research due to its association with metabolic disturbances - most notably insulin resistance (IR). This relationship is multifaceted, involving genetic, biochemical, and pharmacological factors. Numerous studies have consistently shown that IR is more prevalent in individuals with schizophrenia compared to healthy populations (1–3). IR, a critical factor in metabolic syndrome, greatly impacts both physical and mental health. Many antipsychotics commonly used for treating schizophrenia, including quetiapine and olanzapine, are associated with IR and metabolic syndrome (4, 5). A notable incidence of IR is found in first-episode drug-naïve schizophrenia patients, indicating that factors beyond medication might also play a role in IR within this group (6). ​These results suggest that metabolic dysregulation may be an intrinsic aspect of schizophrenia, potentially exacerbated by antipsychotics. Mendelian randomization studies have indicated a probable causal relationship between IR and the risk of schizophrenia (3, 7). Insulin is an essential hormone responsible for regulating glucose and energy balance in the body, especially impacting the brain, which is sensitive to insulin (8, 9). Research suggests that cognitive impairment in schizophrenia may be linked to brain glucose and insulin resistance, indicating that brain insulin resistance could play a role in memory deficits in these patients (10).

In clinical practice, the Homeostasis Model Assessment of Insulin Resistance (HOMA-IR) is commonly used to evaluate insulin sensitivity. The HOMA-IR method assesses insulin sensitivity by dividing the product of fasting insulin and glucose by a constant. In standard clinical practice, its use is limited due to the fact that fasting plasma insulin levels are not commonly assessed (11).

Quantitative computed tomography (QCT) is an imaging technique used to evaluate body composition, including bone mineral density (BMD), liver fat content, visceral adipose tissue (VAT), and subcutaneous adipose tissue (SAT) (12–14). ​These quantitative parameters derived from QCT can be assessed simultaneously during chest, abdominal and/or lumbar exams without additional CT scans, radiation exposure, scan duration, cost, or patient inconvenience. One study suggests that VAT and SAT, measured with QCT, are predictors of metabolic syndrome (15). Evidence from several studies suggests that VAT and BMD are related to IR (12, 14, 16).

At present, the quantitative parameters of QCT for predicting IR in Chinese patients with schizophrenia have not been fully investigated. The connection between BMD, liver fat content, abdominal adiposity, and IR in schizophrenia patients is not yet fully understood. This study investigates the link between the HOMA-IR index and organ body composition, and evaluates the potential of QCT parameters as biomarkers for indicating insulin resistance in schizophrenia.

## Materials and methods

### Participants

Approved by the Institutional Review Board of the Shanghai Mental Health Center, this study was conducted between June 2019 and April 2020. All patients met the DSM-IV criteria for schizophrenia or schizophreniform disorder. Exclusion criteria include: 1) Patients with severe obesity-related comorbidities due to somatic or endocrine disorders; 2) Pregnant, lactating, or planning pregnancy; 3) Inpatients with alcohol or drug dependence as per ICD-10; 4) Patients with thoracolumbar spine metallic implants; 5) Patients with a history of hepatic surgery, significant hepatic lesions, or cirrhosis; 6) Patients unable to specify duration; 7) Patients on lipid-lowering therapy; 8) Patients with hyperthyroidism. In accordance with the specified criteria, a total of 924 subjects with schizophrenia (SCZ) were enrolled.

### Demographic and clinical biochemical parameters

The study utilized the following variables: age (years), gender, body mass index (BMI) (kg/m²), course (years), fasting glucose (mmol/L), fasting insulin (pmol/L), triglycerides (TAG) (mmol/L), cholesterol (Cho) (mmol/L), high-density lipoprotein (HDL) (mmol/L), low-density lipoprotein (LDL) (mmol/L), apolipoprotein A (ApoA) (g/L), apolipoprotein B (ApoB) (g/L), apolipoprotein E (ApoE) (mg/L), lipoprotein A (LppA) (mg/L), T3 (nmol/L), T4 (nmol/L), FT3 (pmol/L), FT4 (pmol/L), and TSH (uIU/ml).The Homeostasis Model Assessment for Insulin Resistance (HOMA-IR) was employed to evaluate insulin resistance status. HOMA-IR quantifies insulin resistance using the formula: fasting insulin (pmol/L) × fasting glucose (mmol/L)/135 (17). A HOMA-IR value > 2.5 was defined as the cutoff threshold for identifying insulin resistance (11). Schizophrenia patients were categorized into non-insulin-resistant (non-IR) and insulin-resistant (IR) groups based on their HOMA-IR values, with thresholds of ≤ 2.5 and > 2.5, respectively.

### QCT examinations and image analysis

Abdominal QCT scans were performed using a 64-slice CT scanner (uCT 760, United Imaging Healthcare, Shanghai, China) alongside a Mindways QCT calibration phantom (Mindways Software Inc., Austin, TX, USA).CT scanning parameters included a tube voltage of 120 kV, tube current of 200 mAs, gantry rotation speed of 0.5 s, pitch of 1.0875 mm, a 512 × 512 matrix, and a scanning field of view of 500 mm. CT image reconstruction utilized a standard algorithm with a slice thickness of 1.5 mm and interslice spacing of 1 mm.

**Figure 1:**
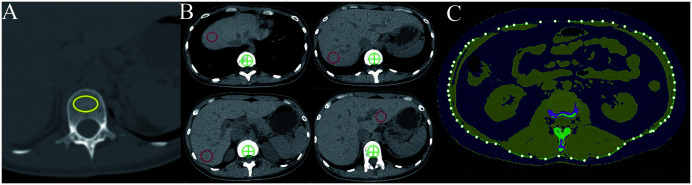
Diagram illustrating the measurement of QCT parameters. **(A)** illustrates the bone mineral density (BMD) region of interest (ROI); **(B)** highlights the liver fat content ROIs; **(C)** displays visceral fat as the blue area within the green line and subcutaneous fat as the blue area outside the green line.

The images were analyzed using the 3D spine function module of QCT Pro Version 6.1 software (Mindways Software Inc., Austin, TX, USA) after being transferred to a QCT bone density measurement workstation. Trabecular bone mineral density (BMD) in the lumbar spine (L1-L2) was measured using QCT. The mean BMD was derived from the two vertebral bodies, excluding areas with fractures, hemangiomas, or focal lytic or sclerotic lesions (**Figure 1A**). To evaluate liver fat content, manually delineated circular regions of interest (ROIs) were positioned on the axial liver slice, with each ROI encompassing an approximate area of 300 mm² while meticulously avoiding bile ducts, vascular structures, and hepatic parenchymal margins (**Figure 1B**).The mean value of these ROIs was calculated to quantify liver fat content. The amount of abdominal subcutaneous fat (SAT) and visceral fat (VAT) was measured at the L2-L3 intervertebral space level (**Figure 1C**).

### Statistical analysis

Data distribution normality and variance homogeneity were assessed using the Kolmogorov-Smirnov and Levene’s tests, respectively. For quantitative variables, comparisons were performed using independent samples t-tests, while categorical variables were analyzed via chi-squared tests. Variables showing statistical significance in the univariate analysis were incorporated into the multivariate logistic regression. ROC curves were generated, and metrics such as AUC, optimal cutoff values, accuracy, sensitivity, specificity, PPV, and NPV were computed. Statistical analyses were performed using R software (version 4.1.3), considering *P* < 0.05 as the threshold for significance.

## Results

### Demographic and clinical biochemical parameters

**Table 1** displays demographic and clinical biochemical characteristics. Differences were observed in age, BMI, course, fasting glucose, fasting insulin, TAG, Cho, HDL, LDL, ApoA, ApoE, and T4 between the non-IR and IR groups, while no differences were found in gender, ApoB, LppA, T3, FT3, FT4, and TSH.

**Table 1:** Demographic and clinical biochemical features of individuals with schizophrenia categorized by insulin resistance status.

Variable	HOMA-IR ≤2.5	HOMA-IR >2.5	t or χ²	*P* value
Age (years)	62.43 ± 12.50	59.73 ± 13.30	3.07	0.002
Gender				
male	406	213	1.88	0.17
female	186	119		
BMI (kg/m²)	21.91 ± 3.23	25.94 ± 3.59	-17.49	<0.001
Course (years)	35.55 ± 13.71	32.77 ± 13.79	2.94	0.003
Fasting glucose (mmol/L)	5.01 ± 1.22	6.71 ± 2.81	-10.49	<0.001
Fasting insulin (pmol/L)	36.76 ± 16.72	129.19 ± 162.92	-10.31	<0.001
Triglycerides (mmol/L)	1.08 ± 0.53	1.84 ± 1.32	-9.92	<0.001
Cholesterol (mmol/L)	4.03 ± 0.91	4.41 ± 0.98	-6.04	<0.001
HDL (mmol/L)	1.20 ± 0.34	1.07 ± 0.31	5.59	<0.001
LDL (mmol/L)	2.65 ± 0.72	3.02 ± 0.74	-7.47	<0.001
Apolipoprotein A (g/L)	1.15 ± 0.23	1.12 ± 0.20	2.17	0.030
Apolipoprotein B (g/L)	0.86 ± 0.23	0.85 ± 0.21	0.11	0.912
Apolipoprotein E (mg/L)	37.62 ± 16.63	43.04 ± 22.92	-3.78	<0.001
Lipoprotein A (mg/L)	188.67 ± 164.21	172.33 ± 172.76	1.40	0.161
T3 (nmol/L)	1.57 ± 5.04	1.44 ± 0.33	0.47	0.639
T4 (nmol/L)	97.71 ± 19.94	94.54 ± 19.76	2.33	0.020
FT3 (pmol/L)	3.95 ± 2.88	4.05 ± 0.67	-0.63	0.530
FT4 (pmol/L)	15.91 ± 4.05	15.62 ± 3.02	1.13	0.258
TSH (uIU/ml)	3.79 ± 3.78	5.22 ± 25.15	-1.03	0.303

HDL refers to High-density lipoprotein and LDL to Low-density lipoprotein. Data are presented as mean ± SD or as counts.

### QCT quantitative parameters

**Figure 2:**
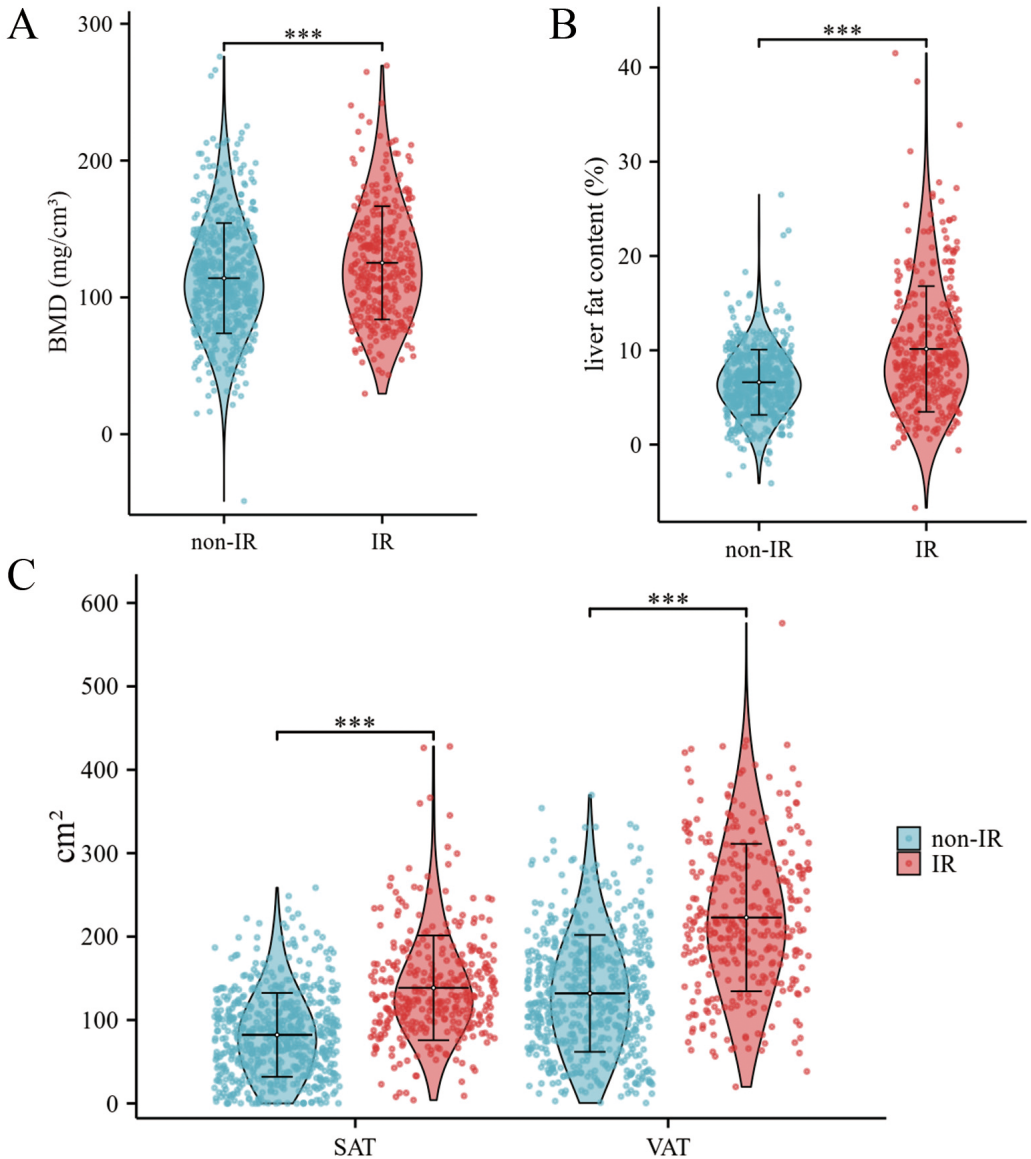
Three violin plots compare non-insulin resistant (non-IR) and insulin resistant (IR) groups. Plot **(A)** shows bone mineral density (BMD), with IR having slightly higher values. Plot **(B)** depicts liver fat content, showing IR with significantly higher levels. Plot **(C)** presents subcutaneous (SAT) and visceral adipose tissue (VAT) areas, with IR having higher values for both, especially VAT. ***P < 0.001.

**Table 2** presents the QCT quantitative parameters for both the non-IR and IR groups. The IR group demonstrated significantly higher BMD, liver fat content, SAT, and VAT than the non-IR group (**Figure 2**).

**Table 2:** QCT quantitative parameters of individuals with schizophrenia categorized by insulin resistance status.

QCT parameters	HOMA-IR ≤2.5	HOMA-IR >2.5	t value	*P* value
BMD (mg/cm^3^)	114.01 ± 40.33	125.27 ± 41.406	-4.03	<0.001
Liver fat (%)	6.61 ± 3.46	10.14 ± 6.66	-8.99	<0.001
SAT (cm^2^)	82.20 ± 50.30	138.55 ± 62.78	-14.02	<0.001
VAT (cm^2^)	131.92 ± 70.04	222.83 ± 88.37	-16.12	<0.001

QCT stands for Quantitative Computed Tomography, BMD for Bone Mineral Density, SAT for Subcutaneous Adipose Tissue, and VAT for Visceral Adipose Tissue. Data are presented as mean ± standard deviation.

### Indicative capability of QCT quantitative parameters for insulin resistance status

Univariate logistic regression analysis indicated that BMD, liver fat content, SAT, and VAT were significantly associated with IR risk. The multivariate analysis (**Table 3**) confirmed these factors as independent risk factors for IR. However, after adjustment for age, sex, and BMI in a subsequent model, only VAT and liver fat content remained statistically significant independent indicators of IR (**Table 4**). SAT was no longer an independent risk factor after accounting for these covariates.

**Table 3:** Logistic regression analysis of the QCT quantitative parameters to identify factors affecting insulin resistance.

Parameters	Univariate analysis		Multivariate analysis	
	OR (95% CI)	*P* value	OR (95% CI)	*P* value
BMD (mg/cm^3^)	1.007 (1.003 – 1.010)	< 0.001	1.006 (1.002 – 1.011)	0.002
Liver fat (%)	1.165 (1.127 – 1.204)	< 0.001	1.101 (1.060 – 1.143)	< 0.001
SAT (cm^2^)	1.019 (1.016 – 1.022)	< 0.001	1.012 (1.008 – 1.015)	< 0.001
VAT (cm^2^)	1.015 (1.012 – 1.017)	< 0.001	1.010 (1.008 – 1.012)	< 0.001

BMD refers to Bone Mineral Density, SAT to Subcutaneous Adipose Tissue, VAT to Visceral Adipose Tissue, CI to Confidence Interval, and OR to Odds Ratio.

**Table 4:** Multivariable logistic regression analysis for insulin resistance adjusted for clinical covariates.

Parameters	Adjusted OR (95% CI)	*P* value
BMD (mg/cm^3^)	1.004 (0.999 – 1.008)	0.154
Liver fat (%)	1.092 (1.051 – 1.135)	< 0.001
SAT (cm^2^)	1.004 (1.000 – 1.008)	0.062
VAT (cm^2^)	1.013 (1.009 – 1.016)	< 0.001
Gender (female)	2.496 (1.638 – 3.802)	< 0.001
age	0.970 (0.954 – 0.987)	< 0.001
BMI	1.102 (1.018 – 1.192)	0.016

This table presents the results of the model adjusting for key clinical variables. VAT and Liver fat content remained significant independent predictors.

**Figure 3:**
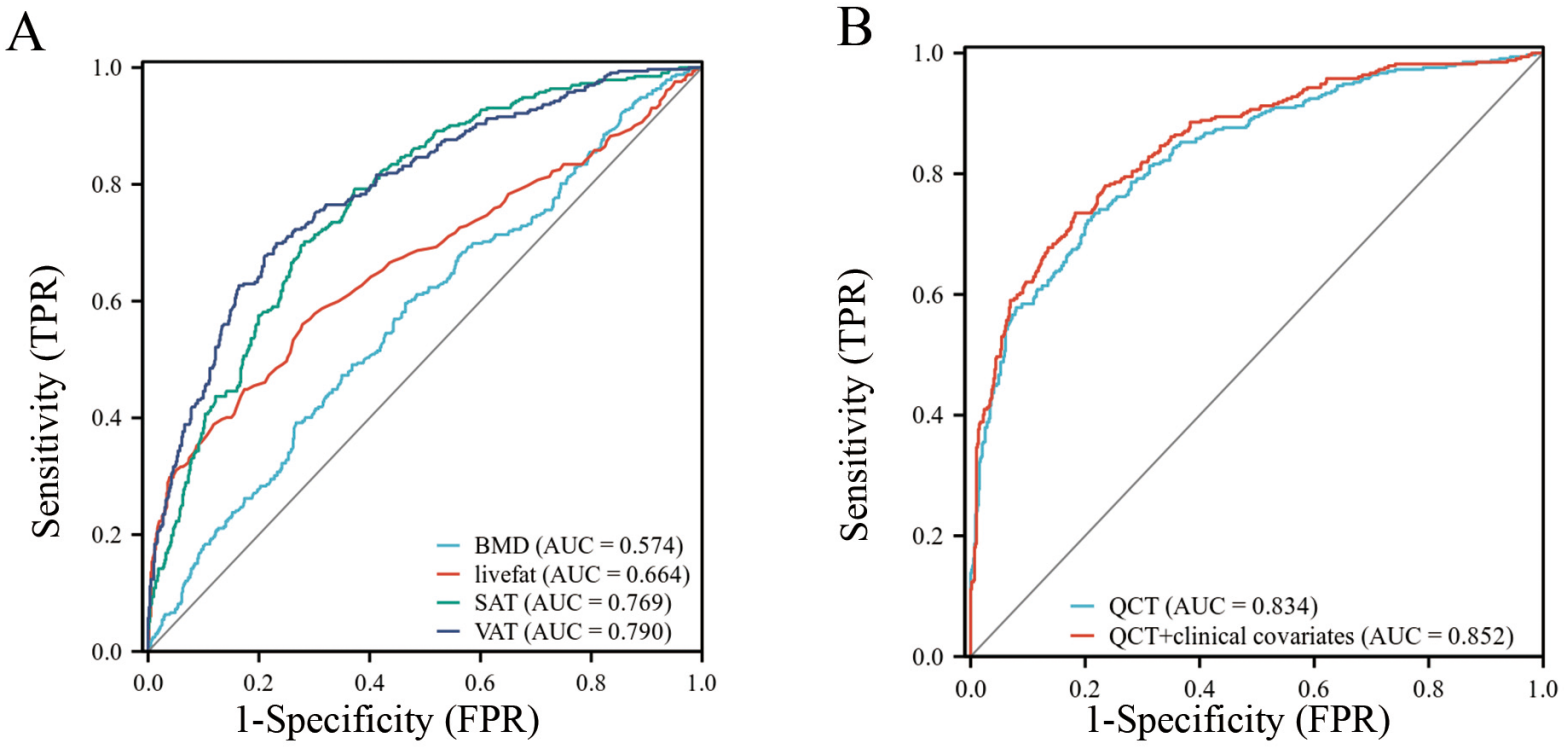
Two ROC curve graphs comparing performance. Graph **(A)** shows curves for BMD, livefat, SAT, and VAT, with AUCs of 0.574, 0.664, 0.769, and 0.790, respectively. Graph **(B)** shows curves for QCT and QCT plus clinical covariates, with AUCs of 0.834 and 0.852, respectively. Both graphs plot sensitivity (TPR) against 1-specificity (FPR).

ROC analysis indicated that VAT achieved an AUC of 0.790 (95% CI: 0.760-0.821), SAT had an AUC of 0.769 (95% CI: 0.738-0.800), liver fat content recorded an AUC of 0.664 (95% CI: 0.625-0.703), and BMD showed an AUC of 0.574 (95% CI: 0.536-0.613) (**Table 5**, **Figure 3A**). The logistic regression model based on QCT parameters achieved a higher AUC of 0.834 (95% CI: 0.807–0.862) for indicating insulin resistance. Moreover, the model incorporating both QCT parameters and clinical covariates attained the highest AUC of 0.852 (95% CI: 0.825–0.878) (**Table 5**, **Figure 3B**). DeLong’s test indicated that the difference in AUC between the QCT parameters-only model and the QCT parameters and clinical covariates model was statistically significant (*P* = 0.001). This finding demonstrated that the addition of clinical covariates (gender, age, BMI) significantly improved the indicative performance of the model based on QCT parameters.

**Table 5:** Predictive capability of QCT quantitative parameters for insulin resistance status.

Variable	AUC (95% CI)	Cutoff value	Youden index	Sen (%)	Spe (%)	PPV (%)	NPV (%)	Acc (%)
BMD (mg/cm^3^)	0.574 (0.536 – 0.613)	113.65	0.132	59.64	53.54	41.86	70.29	55.74
Liver fat (%)	0.664 (0.625 – 0.703)	8.15	0.282	56.02	72.13	52.99	74.52	66.34
SAT (cm^2^)	0.769 (0.738 – 0.800)	106	0.420	70.18	71.79	58.25	81.11	71.21
VAT (cm^2^)	0.790 (0.760 – 0.821)	179.55	0.467	69.88	76.86	62.87	81.98	74.35
Combined (QCT)	0.834 (0.807 – 0.862)	-0.50	0.519	73.49	78.38	65.59	84.06	76.62
Combined (QCT+clinical covariates)	0.852 (0.825 – 0.878)	-0.44	0.553	73.49	81.76	69.32	84.62	78.79

Sen represents Sensitivity, Spe stands for Specificity, PPV denotes positive predictive value, NPC signifies negative predictive value, and Acc indicates accuracy.

**Figure 4:**
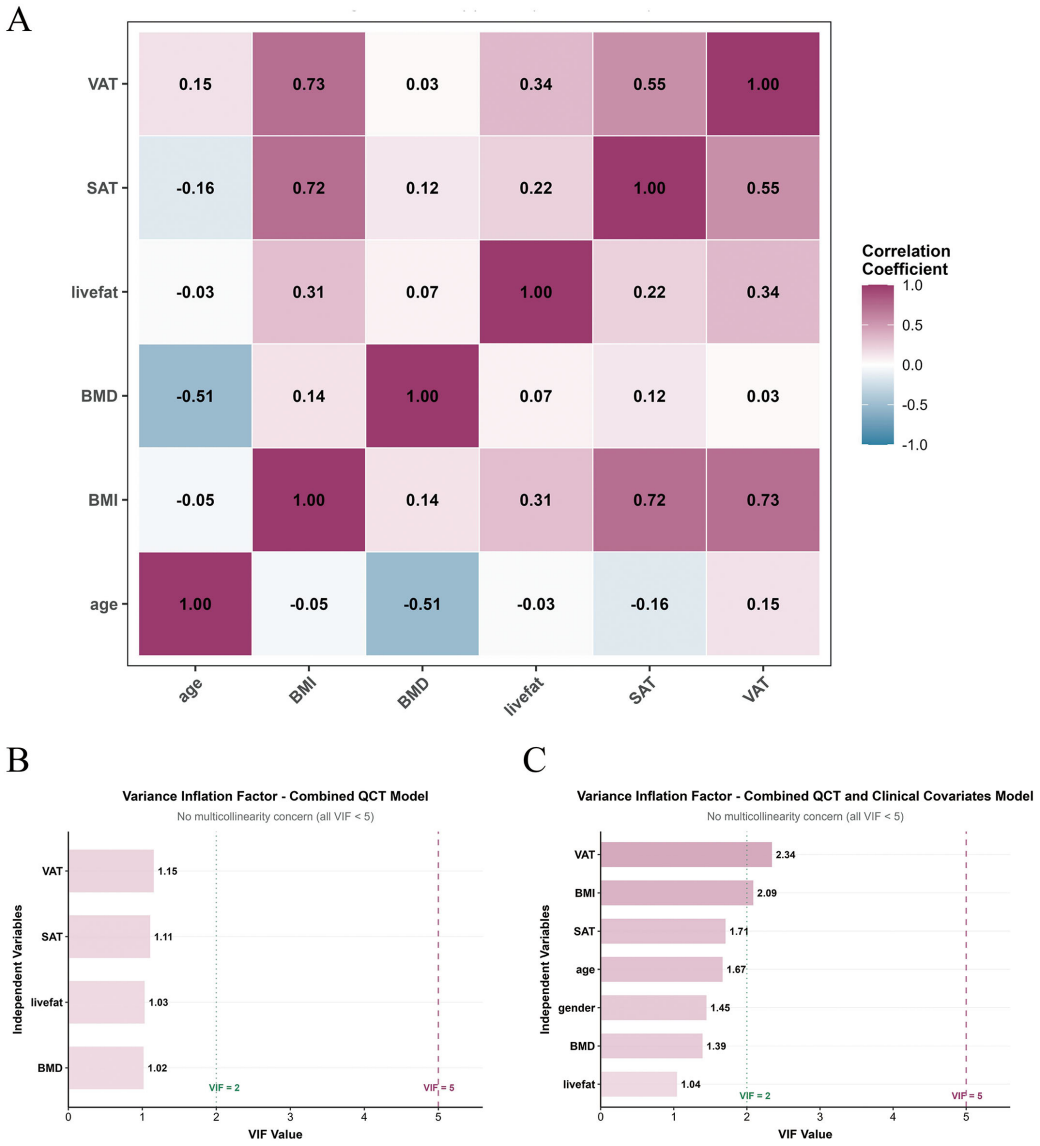
Panel **(A)** shows a correlation matrix of variables, including VAT, SAT, livefat, BMD, BMI, and age, using a color gradient to indicate correlation strength. Panels **(B, C)** display variance inflation factor (VIF) bar charts for the Combined QCT Model and Combined QCT and Clinical Covariates Model, charts highlight no multicollinearity concern as all VIF values are below 5.

To validate the rationality of variable selection in the indicative models, we first examined pairwise relationships between continuous variables. The correlation matrix for the combined model (**Figure 4A**) showed no strong linear correlations (|r| > 0.8) among off-diagonal variables, preliminarily supporting variable independence and providing a basis for further multicollinearity assessment. We then performed variance inflation factor (VIF) analysis to systematically evaluate multicollinearity, using a VIF threshold of 5 to indicate significant collinearity. In the QCT parameters only model, all VIF values were below 5 (**Figure 4B**), indicating no substantial multicollinearity and supporting model stability and reliability. Similarly, in the combined model incorporating QCT parameters and clinical covariates, all variables also had VIF values below 5 (**Figure 4C**), confirming that adding clinical covariates did not introduce severe multicollinearity.

## Discussion

Our analysis demonstrated that patients with insulin resistance (IR) exhibited significantly higher bone mineral density (BMD), liver fat content, subcutaneous adipose tissue (SAT), and visceral adipose tissue (VAT) compared to those without IR (all *P* < 0.001). Univariate and multivariate logistic regression analyses, incorporating only QCT parameters, identified all four parameters as significant factors associated with IR. However, after adjustment for clinical covariates—gender, age, and body mass index (BMI)—only VAT and liver fat content remained statistically significant independent predictors. This finding suggests that the apparent association between SAT and IR is substantially confounded by these clinical factors, and highlights that the contribution of adipose tissue to IR in schizophrenia is primarily driven by its ectopic deposition in specific depots, particularly the visceral compartment and the liver. Furthermore, ROC curve analysis underscored the clinical utility of these measures: a combined model integrating QCT parameters with clinical covariates yielded the highest predictive accuracy for IR, with an AUC of 0.852 (95% CI: 0.825–0.878).

Insulin secreted by pancreatic β-cells is essential for glucose homeostasis (18). Insulin resistance results in elevated circulating insulin concentrations as a compensatory mechanism to maintain normoglycemia and prevent hyperglycemia. Insulin, an anabolic hormone, is crucial for maintaining and improving bone mineral density and structural integrity (19, 20). Conversely, bone tissue may modulate the body’s sensitivity to insulin (16, 20, 21). Recent research indicates that bones, beyond their structural support role, function as an endocrine organ influencing energy metabolism. Osteoblasts, responsible for bone synthesis and mineralization, possess insulin receptors that, upon activation by insulin, trigger postnatal bone acquisition and enhance osteocalcin production, which subsequently influences pancreatic insulin secretion to maintain glucose homeostasis (20). Osteoclasts may contribute to glucose uptake-associated insulin resistance through the secretion of resistin (21). The concept that interfering with bone resorption adversely affects glucose balance is crucial in the medical field, given that most osteoporosis medications target this bone remodeling process (22). Studies on various populations have explored the link between insulin resistance and BMD, but the results have been inconsistent. In non-diabetic Caucasian menopausal women, both total hip and lumbar BMD showed a significant positive correlation with HOMA-IR, which persisted after adjusting for body weight (23). In a study involving South Korean men, HOMA-IR was found to have a significant negative correlation with BMD (24). In healthy, non-obese, menopausal Chinese-Singaporean women, an inverse association was found between HOMA-IR and lumbar spine (L1–L4) BMD, even after controlling for lean body mass and age (16). Our study revealed that although patients in the IR group exhibited significantly higher BMD in univariate analysis, this association lost significance after adjusting for age, gender, and BMI. This finding provides important insight into the complex interplay between bone and energy metabolism. The loss of independent significance of BMD suggests that its apparent association with IR is likely not a direct causal relationship, but rather mediated or confounded by other factors.

Liver fat content and insulin resistance play key roles in the development of metabolic disorders like nonalcoholic fatty liver disease (NAFLD) and type 2 diabetes mellitus (T2DM). The interplay between hepatic fat content and insulin resistance is intricate, with evidence indicating that elevated hepatic lipid accumulation drives insulin resistance, which in turn exacerbates hepatic steatosis. Metformin, a well-known antidiabetic medication, has been shown to significantly reduce liver fat content in patients with schizophrenia experiencing olanzapine-induced weight gain (25). The study identified a positive correlation between liver fat changes and HOMA-IR, indicating that metformin’s influence on liver fat might be mediated by its effect on insulin resistance (25). In patients newly diagnosed with T2DM, elevated liver fat content was strongly correlated with increased HOMA-IR, highlighting a significant association between hepatic steatosis and insulin resistance. This study also suggested that liver fat content could serve as a potential indicator for the prediction of T2DM, emphasizing the importance of monitoring liver fat in at-risk populations (26). The liver-alpha cell axis, involving liver fat and glucagon interactions, correlates with HOMA-IR, highlighting the complex relationship between liver fat content and insulin resistance (27). Our results aligned with previous research, indicating hepatic fat accumulation is a key risk factor for insulin resistance in schizophrenia patients.

Research indicates that different fat compartments affect insulin sensitivity and lipid levels differently, with excess visceral adipose tissue (VAT) posing a greater risk for insulin resistance and hyperlipidemia compared to excess subcutaneous adipose tissue (SAT) (28, 29). Research indicates that adipose tissue releases mediators like TNF-α, IL-6, leptin, and adiponectin, which affect insulin function in target tissues (30). The Framingham Heart Study indicates that increased volumes of VAT and SAT negatively correlate with total adiponectin and resistin levels, while positively associating with systemic inflammation markers like IL-6 and C-Reactive protein (31, 32). Uncoupling protein 1 (UCP1) expression is notably higher in visceral adipose tissue (VAT) than in subcutaneous adipose tissue (SAT), with a negative correlation observed between UCP1 expression in SAT and HOMA-IR (33). In human white adipocytes, UCP1 mRNA expression is downregulated by pro-inflammatory stimuli while being upregulated by metabolically enhancing agents (33). The Dallas Heart study identified a positive correlation between VAT, SAT, and HOMA-IR in univariable analyses. In multivariable analyses, VAT still showed an association with insulin resistance markers, but the link with SAT was not significant after adjustment for BMI (34). Unlike the general population, the link between SAT/VAT and insulin resistance remains unexplored in individuals with schizophrenia. In our study, the loss of independent predictive value by SAT after adjustment for gender, age, and BMI is highly informative. This suggests that the risk associated with general adiposity (as captured by BMI) and subcutaneous fat may be largely mediated by its correlation with the more metabolically active visceral and hepatic fat. Therefore, our study highlights that VAT and liver fat, as measured by QCT, are the core adiposity-related drivers of insulin resistance in this patient population, providing distinct information beyond BMI.

Despite its strengths, this study is not without limitations. First, its cross-sectional and single-center design precludes establishing a causal relationship between body composition and insulin resistance, thereby necessitating validation in longitudinal studies. Second, the absence of a healthy control group impedes our ability to determine whether the roles of VAT and liver fat are specific to schizophrenia or reflect a general metabolic pathway. Furthermore, we were unable to fully adjust for key confounding factors, including specific antipsychotic treatment regimens, lifestyle variables (e.g., dietary patterns and physical activity), and inflammatory biomarkers. To confirm our findings and clarify the underlying mechanisms, future prospective studies—incorporating healthy control groups and rigorously collecting these clinical and biochemical data—are therefore warranted.

In conclusion, among the QCT parameters evaluated, visceral adipose tissue and liver fat content were identified as the most robust and independent predictors of insulin resistance in patients with schizophrenia, even after accounting for age, sex, and BMI. This underscores the critical importance of ectopic fat deposition in the pathogenesis of metabolic disturbances in this vulnerable population.

## Data Availability

The raw data supporting the conclusions of this article will be made available by the authors, without undue reservation.
